# Dietary Modification Alters the Intrarenal Immunologic Micromilieu and Susceptibility to Ischemic Acute Kidney Injury

**DOI:** 10.3389/fimmu.2021.621176

**Published:** 2021-03-11

**Authors:** Junseok Jeon, Kyungho Lee, Kyeong Eun Yang, Jung Eun Lee, Ghee Young Kwon, Wooseong Huh, Dae Joong Kim, Yoon-Goo Kim, Hye Ryoun Jang

**Affiliations:** ^1^Division of Nephrology, Department of Medicine, Samsung Medical Center, Sungkyunkwan University School of Medicine, Seoul, South Korea; ^2^Division of Scientific Instrumentation and Management, Korea Basic Science Institute, Daejeon, South Korea; ^3^Department of Pathology, Samsung Medical Center, Sungkyunkwan University School of Medicine, Seoul, South Korea

**Keywords:** diet, high-salt diet, high-fat diet, acute kidney injury, immunologic micromilieu, ischemia-reperfusion injury

## Abstract

The versatility of the intrarenal immunologic micromilieu through dietary modification and the subsequent effects on susceptibility to ischemic acute kidney injury (AKI) are unclear. We investigated the effects of high-salt (HS) or high-fat (HF) diet on intrarenal immunologic micromilieu and development of ischemic AKI using murine ischemic AKI and human kidney-2 (HK-2) cell hypoxia models. Four different diet regimens [control, HF, HS, and high-fat diet with high-salt (HF+HS)] were provided individually to groups of 9-week-old male C57BL/6 mice for 1 or 6 weeks. After a bilateral ischemia-reperfusion injury (BIRI) operation, mice were sacrificed on day 2 and renal injury was assessed with intrarenal leukocyte infiltration. Human kidney-2 cells were treated with NaCl or lipids. The HF diet increased body weight and total cholesterol, whereas the HF+HS did not. Although the HF or HS diet did not change total leukocyte infiltration at 6 weeks, the HF diet and HF+HS diet increased intrarenal CD8 T cells. Plasma cells increased in the HF and HS diet groups. The expression of proinflammatory cytokines including TNF-α, IFN-γ, MCP-1, and RANTES was increased by the HF or HS diet, and intrarenal VEGF decreased in the HS and HF+HS diet groups at 6 weeks. Deterioration of renal function following BIRI tended to be aggravated by the HF or HS diet. High NaCl concentration suppressed proliferation and enhanced expression of TLR-2 in hypoxic HK-2 cells. The HF or HS diet can enhance susceptibility to ischemic AKI by inducing proinflammatory changes to the intrarenal immunologic micromilieu.

## Introduction

Ischemic acute kidney injury (AKI) is the most common cause of AKI and frequently contributes to development and progression of chronic kidney disease (CKD) in both native and transplanted kidneys (1, 2). Ischemia-reperfusion injury (IRI) induces graft injury, an inevitable consequence of kidney transplantation (3). Substantial roles of immunologic mechanisms, beyond simple hypoxic injury, in the pathogenesis of ischemic AKI have been demonstrated in many studies (4, 5). In the post-ischemic kidney following IRI, a robust inflammatory response caused by both innate and adaptive immune systems results in kidney damage (6). In addition to infiltration of circulating immune cells, the intrarenal immunologic micromilieu, including resident intrarenal immune cells (7) and Toll-like receptors (TLRs) on renal tubules (8), contributes significantly to renal injury following IRI (6).

Recent studies have reported a relationship between diet and immune function (9). Changes in dietary composition have the potential to exacerbate or alleviate the severity of diseases in which immune mechanisms play an important role in the pathogenesis, such as hypertension in a murine model of salt-sensitive hypertension or obesity-related kidney damage in a high-fat (HF) diet-induced obesity model (10–12). However, it is yet to be determined whether dietary modification can alter the intrarenal immunologic micromilieu and susceptibility to ischemic AKI, although the role of dietary intervention in CKD has been reported (13). Considering the potential for preventive or therapeutic effects of dietary intervention in ischemic AKI, it is important to investigate the effects of dietary modification in normal kidneys.

In this study, we aimed to reveal the effects of HF or high-salt (HS) diet on normal kidneys and development of ischemic AKI with a focus on the intrarenal immunologic micromilieu.

## Materials and Methods

### Dietary Modification and the Renal IRI Model

This study was approved by the Samsung Medical Center Animal Care and Use Committee and the Institutional Review Board of Samsung Medical Center (IACUC No. 20180314002) and was performed in compliance with the animal research: reporting *in vivo* experiments guidelines (14, 15). Male 9-week-old C57BL/6 mice were purchased from Orient Bio Inc. (Seongnam, Kyoungki-do, Korea). All mice were housed in a specific pathogen-free barrier facility.

We investigated the effects of HF or HS diet on normal kidney and post-ischemic kidneys in an ischemic AKI model induced by bilateral IRI surgery. In each model, mice were randomly allocated into four diet regimens; normal diet (0.25% NaCl by weight, 10% fat by calories), HF diet (0.25% NaCl by weight, 60% fat by calories), HS diet (8% NaCl by weight, 10% fat by calories), and high-fat diet with high-salt (HF+HS) (8% NaCl by weight, 60% fat by calories). The composition of the normal and HS diets was 20% protein (main source casein), 70% carbohydrate (main source sucrose), and 10% fat (main source soybean oil) (D12450B, Research Diets, New Brunswick, NJ). The composition of the HF diet and the HF+HS diet was 20% protein (main source casein), 20% carbohydrate (main source Lodex 10), and 60% fat (main source lard) (D12492, Research Diets). All diet regimens had the same mineral and vitamin concentrations except for NaCl.

As for normal mice, each group was maintained on the allocated diet for 42 days and then switched to a normal diet for 14 days.

Regarding the ischemic AKI model, we used an established murine IRI model with a laparotomy approach (16, 17). Bilateral IRI was induced after 1-week or 6-week durations of dietary modification. Briefly, mice were anesthetized with an intraperitoneal injection of ketamine (100 mg/kg; Yuhan, Seoul, Korea) and xylazine (10 mg/kg; Bayer, Leverkusen, Germany). After an abdominal midline incision, both renal pedicles were isolated and clamped for 27 min with a microvascular clamp (Roboz Surgical Instrument, Gaithersburg, MD). During the operation, anesthetized mice were kept well-hydrated with warm sterile saline and placed on a thermostatically controlled heating table. After 27 min, the microvascular clamps were released from the renal pedicles for reperfusion. After applying sutures, mice were allowed to recover with free access to the allocated diet and water. All mice were sacrificed on day 2 after the IRI operation, and post-ischemic kidneys were harvested after exsanguination.

### Assessment of Renal Function

In normal mice, blood urea nitrogen (BUN; Fujifilm, Bedford, UK) and plasma creatinine (Arbor Assays, Ann Arbor, MI) concentrations were measured in plasma samples collected from tail veins serially on days 0, 7, 14, 28, 42, and 56 after dietary modification. Colorimetric kits were used according to the manufacturer's recommended methods. In the IRI model, plasma samples were measured on days 0, 1, and 2 after the operation using the same methods.

### Tissue Histological Analysis

In the IRI model, post-ischemic kidney tissue sections were fixed with 10% buffered formalin and stained with hematoxylin and eosin. A renal pathologist who was blinded to the diet allocation scored renal tubular necrosis in the cortex and outer medulla of post-ischemic kidneys.

### CD45 Immunohistochemistry and TissueFAXS Analysis

Formalin-fixed renal tissue sections were immunostained for detection of CD45 as follows. Sections (4-μm-thick) were deparaffinized with xylene, rehydrated in a graded alcohol series, and placed in a citrate buffer solution (pH 6.0). Slides were placed in a pressure cooker and heated for 10 min to enhance antigen retrieval. After cooling, the kidney sections were immersed in a hydrogen peroxide solution (Dako, Carpinteria, CA) for 30 min to block endogenous peroxidase activity, followed by overnight incubation at 4°C with serum-free protein block (Dako). The next day, the slides were incubated with a 1:100 dilution of anti-mouse CD45 monoclonal antibody (BD Biosciences, San Jose, CA) for 1 h at room temperature. After being rinsed, the CD45-stained sections were incubated for 30 min at room temperature with a secondary antibody using a Dako REAL EnVison kit (Dako). Subsequently, 3,3′-diaminobenzidine tetrahydrochloride (Dako) was applied to the slides to produce a brown color, and the slides were counterstained with Mayer's hematoxylin solution (Dako).

A TissueFAXS workstation (Tissue Gnostics, Vienna, Austria) was used to analyze and calculate the percentage of CD45-positive cells in kidney samples, as described previously (18).

### Flow Cytometric Analysis of Kidney-Infiltrating Mononuclear Cells

Isolation of kidney mononuclear cells (KMNCs) was based on an established protocol (19). Briefly, decapsulated kidneys were immersed in RPMI buffer (Mediatech, Manassas, VA) containing 5% FBS and disrupted mechanically using a Stomacher 80 Biomaster (Seward, Worthing, UK). Samples were strained, washed, and resuspended in 36% Percoll (Amersham Pharmacia Biotech, Piscataway, NJ) followed by gentle overlaying onto 72% Percoll. The samples were centrifuged at 1,000*g* for 30 min at room temperature. KMNCs were collected from the interface of 36% and 72% Percoll.

Isolated KMNCs were resuspended in FACS buffer and pre-incubated with anti-CD16/CD32 antibodies for 10 min to minimize non-specific binding through Fc-receptors. KMNCs were incubated with anti-mouse anti-CD3, CD4, CD8, CD19, CD21, CD25, CD44, CD45, CD62L, CD69, CD126, CD138, Gr-1, F4/80, FoxP3, and NK1.1 (all from BD Biosciences, San Jose, CA) for 25 min at 4°C, washed with FACS buffer, and fixed with 1% paraformaldehyde solution. Samples were acquired using a BD FACSVerse flow cytometer. Data were analyzed using the FACSuite program (BD Biosciences).

### Multiplex Cytokine/Chemokine Assay

Multiplex cytokine and chemokine analysis in whole kidney protein extracts was conducted using a Milliplex MAP Mouse Cytokine/Chemokine Kit (Luminex, Austin, TX) following the manufacturer's instructions. Anti-cytokine monoclonal antibodies linked to microspheres incorporating distinct properties of two fluorescent dyes were used in this multiplexed particle-based flow cytometric assay. Our assay was designed to quantify interleukin (IL)-2; IL-4; IL-6; IL-10; interferon (IFN)-γ; monocyte chemoattractant protein (MCP)-1; regulated on activation, normal T cell expressed and secreted (RANTES/CCL5); tumor necrosis factor (TNF)-α; and vascular endothelial growth factor (VEGF). The value of each cytokine or chemokine was normalized by dividing the raw concentration (pg/ml) by the kidney protein concentration (mg/ml, measured by using a Pierce BCA protein assay kit, Thermo Fisher Scientific, Waltham, MA).

### Western Blot Analysis of Intrarenal Toll-Like Receptors 2 and 4

Intrarenal Toll-like receptors 2 and 4 (TLR-2 and TLR-4) were analyzed by western blot analysis. According to the manufacturer's instructions, equal amounts of whole kidney protein extract (30 μg) were separated by electrophoresis on a NuPAGE Bolt mini gel system (Thermo Fisher Scientific). The gels were transferred onto a nitrocellulose membrane using an iBlot 2 Dry Blotting System (Thermo Fisher Scientific) after electrophoresis. Membranes were blocked with 5% skim milk tris-buffered saline solution with 0.1% Tween20 (TBST) for 1 h at room temperature and then incubated overnight at 4°C with one of the following antibodies: mouse monoclonal anti-TLR2 antibody (MyBioSource, San Diego, CA) or anti-TLR4 antibody (Novus Biologicals, Centennial, CO). The horseradish peroxidase-conjugated secondary antibody was applied for 30 min at room temperature after washing with TBST. The signal was visualized using an Amersham ECL detection system (GE Healthcare, Chicago, IL), following the manufacturer's instructions. Bands were densitometrically analyzed using ImageJ 1.8 software (Wayne Rasband, National Institutes of Health, MD) and normalized against corresponding β-actin band intensity as an internal control.

### HK-2 Cell Hypoxia Model and Proliferation Assay

Human kidney-2 (HK-2) cell (an immortalized proximal tubule epithelial cell line from a normal adult human kidney) hypoxia model was used for *in vitro* study to investigate the effects of a HS or HF environment at a cellular level.

Human kidney-2 (HK-2) cells were purchased from the American Type Culture Collection (CRL-2190, Manassas, VA) and cultured in keratinocyte serum-free media (Thermo Fisher Scientific) supplemented with bovine pituitary extract and human recombinant epidermal growth factor. Cells were incubated at 37°C in a humidified atmosphere of 5% CO_2_ with the media changed every 2–3 days. Hypoxia was induced by exposure to 1% O_2_ and 5% CO_2_ balanced with nitrogen in a multi-gas incubator (APM-30D, Astec, Fukuoka, Japan) for 48 h.

HK-2 cells were divided into four groups. The first and second groups were controls under normoxia (21% O_2_) and hypoxia (1% O_2_), respectively. The third and fourth groups were treated with additional NaCl 25 mM and 1:250 diluted lipid concentrate (Thermo Fisher Scientific), respectively, before and after hypoxic insult.

The degree of HK-2 cell proliferation on days 0, 1, and 2 after hypoxia was assessed with a Cell Titer96 aqueous one solution cell proliferation assay (Promega, Madison, WI) according to the manufacturer's instructions.

For quantification of inflammatory signaling molecule expression in the hypoxic HK-2 cells, TLR-2 and TLR-4 were measured with western blot analysis using mouse monoclonal anti-TLR2 (Santa Cruz Biotechnology, Dallas, TX) and anti-TLR4 (Novus Biologicals, Centennial, CO) antibodies on days 0 and 2. Briefly, the cells were placed in RIPA buffer (Sigma-Aldrich, St. Louis, MO) containing a protease inhibitor cocktail (Sigma-Aldrich). The homogenate was centrifuged at 13,000*g* at 4°C for 10 min, and the supernatant was subjected to the aforementioned western blotting procedures.

### Statistical Analyses

All data were expressed as mean ± standard error of the mean (SEM). Differences between groups or time points were analyzed using the Mann-Whitney *U*-test or two-way analysis of variance (ANOVA) followed by Tukey's *post-hoc* analysis. All statistical analyses were conducted using GraphPad Prism version 8 software (GraphPad Software, La Jolla, CA). *P* values <0.05 were considered statistically significant.

## Results

### Effects of Dietary Modification on Physiologic Changes in Normal Mice

**Figure 1:**
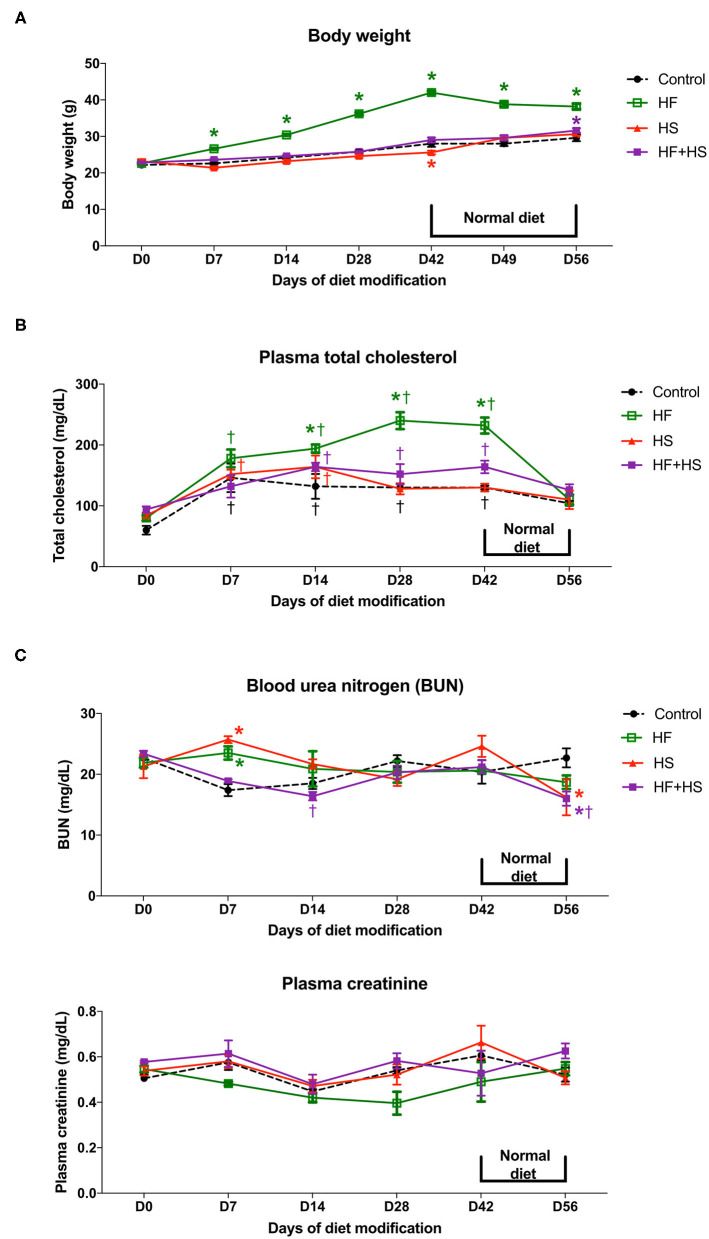
Effects of dietary modification on physiologic changes of normal mice. **(A)** HF diet group gained weight significantly from day 7 after starting the HF diet and showed stationary body weight after switching to a normal diet. The HS diet group had a lower body weight than the control group on day 42 and returned to a body weight comparable with that of the control group 1 week after switching to a normal diet. **(B)** HF diet significantly increased plasma total cholesterol concentration from day 14 after dietary modification. The total cholesterol level of the HF diet group returned to a comparable concentration after switching to a normal diet**. (C)** BUN level on day 7 was significantly higher in the HF diet and HS diet groups. Overall renal function measured by plasma creatinine was comparable among groups for the whole study period.*P < 0.05, compared with the control group at each time point. ^†^*P* < 0.05, compared with day 7 in the same group (*n* = 5 for each group at each time point). Statistical analyses were performed with two-way ANOVA test followed by Tukey's test. HF, high-fat; HS, high-salt; HF+HS, high-fat with high-salt.

To investigate the physiologic changes caused by dietary modification in normal mice, body weight, plasma total cholesterol, creatinine, and BUN concentration were measured serially. The total amount of dietary intake in the HF, HF+HS, and control groups was similar, while the HS group tended to consume slightly more chow (Supplemental Figure 1). The body weight of the HF diet group significantly increased from day 7 after starting the HF diet. The HS diet group had a lower body weight than the control group on day 42 and comparable body weight to that of the control group from 1 week after switching to a normal diet (Figure 1A). Plasma total cholesterol level of the HF diet group was significantly higher compared to that of the control group on day 14 and became comparable to that of the control group at 1 week after switching to a normal diet. Plasma cholesterol level in the control, HS diet, and HF+HS diet groups was comparable during the 6 weeks of dietary modification (Figure 1B). BUN was higher in the HF diet group and the HF+HS diet group on day 7. Overall renal function was comparable among the groups for 6 weeks of dietary modification (Figure 1C).

### Effects of Dietary Modification on the Intrarenal Leukocytes of Normal Mice

**Figure 2:**
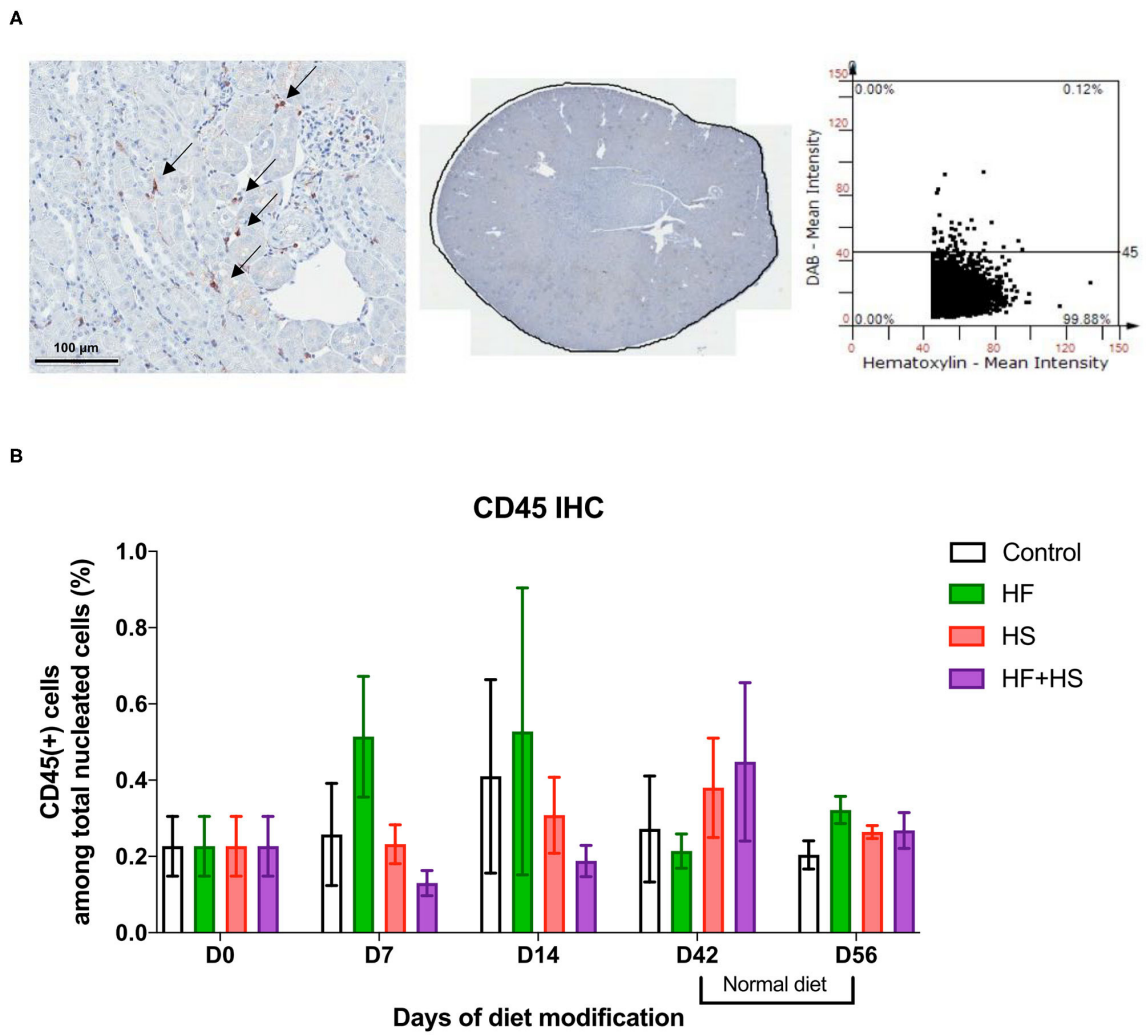
Effects of dietary modification on intrarenal leukocytes of normal mice. Resident leukocytes were analyzed with immunohistochemistry of CD45 and flow cytometry on normal renal tissue. **(A)** Representative immunohistochemistry findings and semiquantitative analysis of CD45-positive leukocytes in normal kidney of the control group on day 0. Arrows indicate CD45-positive leukocytes (×200). **(B)** The percentages of total leukocytes expressing CD45 among total nucleated cells were comparable between diet-fed groups.

Trafficking of total leukocytes into normal kidneys was evaluated by immunohistochemical staining of CD45, followed by semiquantitative analysis with TissueFAXS (Figure 2A). The proportion of intrarenal total leukocytes among total nucleated cells was comparable between groups at each time point (Figure 2B).

**Figure 3:**
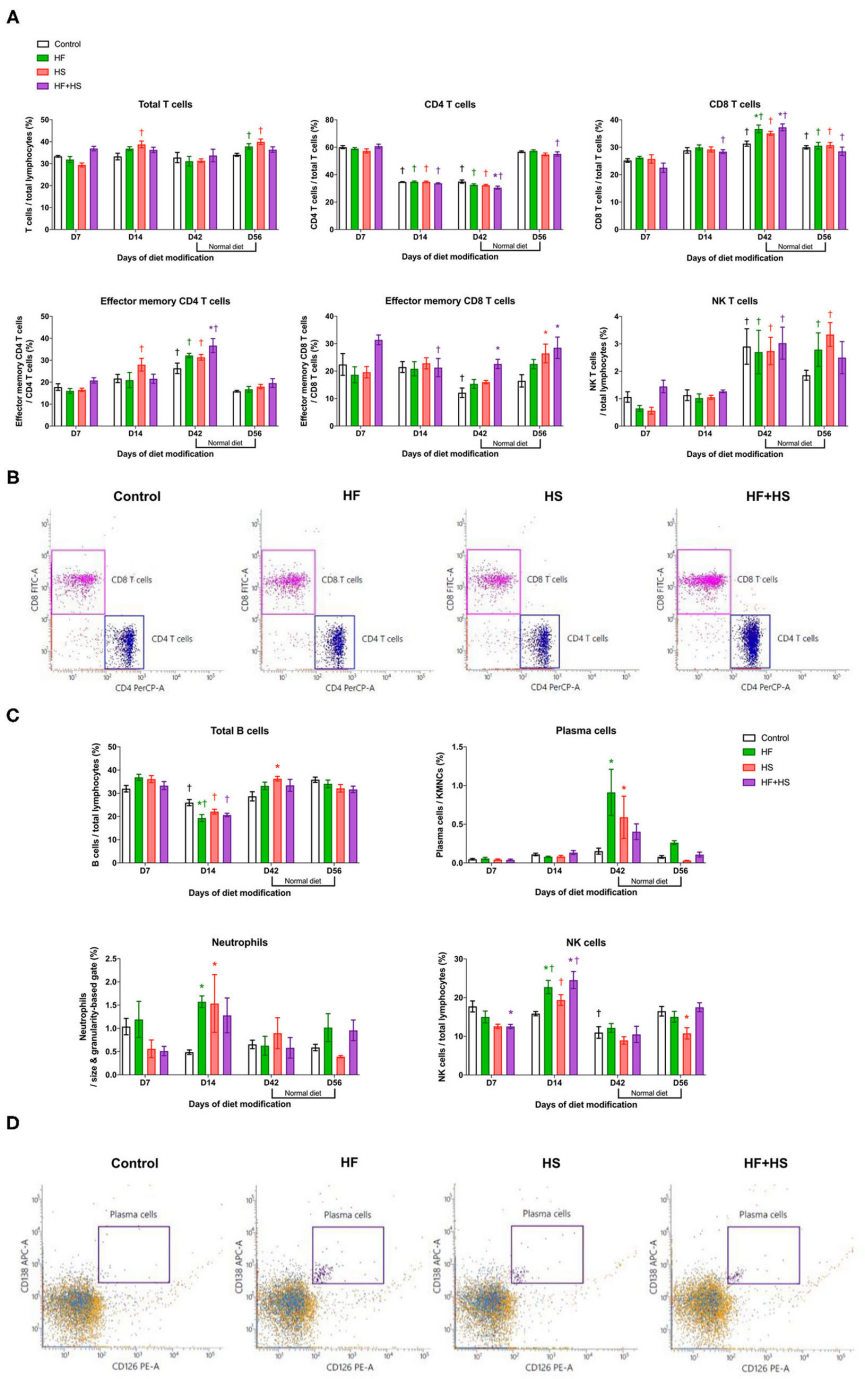
Flow cytometry analyses of KMNCs isolated from kidneys of normal mice. **(A)** Changes in the intrarenal T cell subpopulation by dietary modification. HF and HF+HS diets increased the proportion of total CD8 T cells among total T cells on day 42 after dietary modification. HF+HS diet also increased the proportion of effector memory CD4 and CD8 T cells. **(B)** The representative dot plots analyzing T cells on day 42. Gated cells indicate CD4 T cells and CD8 T cells among total T cells. **(C)** HF diet and HS diet increased the infiltration of plasma cells and neutrophils on days 42 and 14 after dietary modification, respectively. HF diet and HF+HS diet increased the infiltration of activated mature B cells and NK cells on day 14 after dietary modification. **(D)** Representative dot plots analyzing plasma cells on day 42. Gated cells indicate plasma cells expressing CD138 and CD126 among total kidney mononuclear cells. *P < 0.05, compared with the control group at each time point. ^†^*P* < 0.05, compared with day 7 in the same group (*n* = 5 for each group at each time point). Statistical analyses were performed with two-way ANOVA test followed by Tukey's test. HF, high-fat; HS, high-salt; HF+HS, high-fat with high-salt.

Major effector cells of both innate and adaptive immune systems trafficked into the kidneys after dietary modification were analyzed with flow cytometry. Regarding T cell subtype, total CD8 T cells, effector memory CD4 T cells, and NK T cells increased over time during the diet modification, whereas total CD4 T cells decreased. In terms of intergroup differences at each time point, the HF group and the HF+HS group showed a larger proportion of CD8 T cells among total T cells on day 42 after dietary modification compared to the control diet group. The HF+HS group also showed larger proportions of the effector memory subsets of CD4 and CD8 T cells and activated CD4 and CD8 T cells (Figures 3A,B, Supplemental Figure 2). Regarding non-T cell populations, total B cells decreased and NK cells increased on day 14 compared to the day 7 after diet modification. Plasma cells reached peak numbers in the HF diet and HS diet groups on day 42 and decreased to levels comparable with those of the control group after a normal diet for 2 weeks. The proportion of NK cells and activated mature B cells among total B cells were significantly higher in the HF diet and the HF+HS diet groups on day 14 after dietary modification. The HF diet and HS diet groups showed higher intrarenal infiltration of neutrophils on day 14 after dietary modification (Figures 3C,D, Supplemental Figure 2).

### Effects of Dietary Modification on Intrarenal Cytokines/Chemokines of Normal Mice

**Figure 4:**
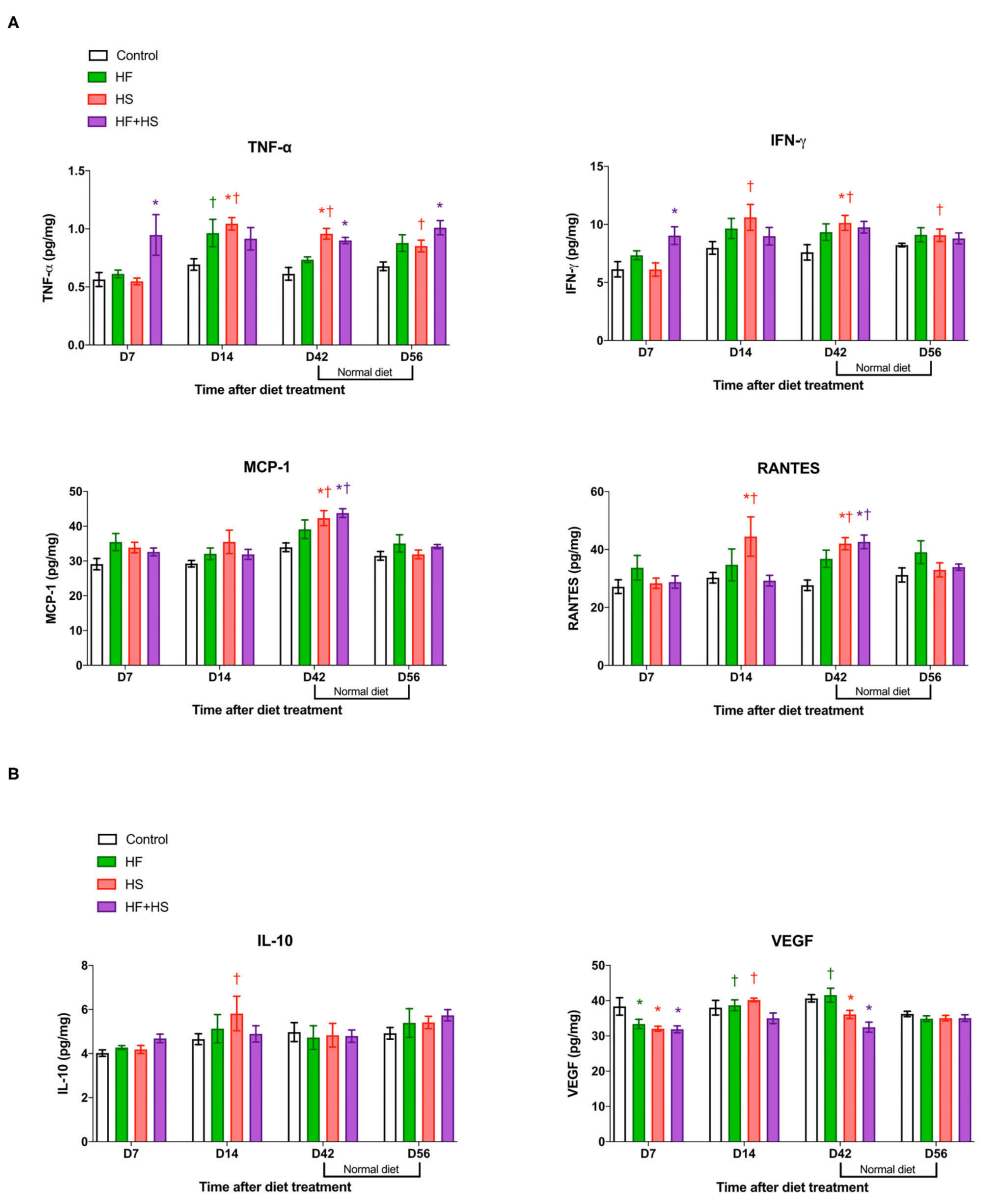
Effects of dietary modification on intrarenal cytokines and chemokines of normal mice. HS and HF+HS diets increased intrarenal expressions of TNF-α, INF-γ, MCP-1, and RANTES on day 42. The HF+HS diet also increased the expression of IL-6 on day 7 after dietary modification. Although there were no significant differences in the expression of IL-10, the intrarenal expression of VEGF was lower in the HS diet group and the HF+HS diet group on day 7 and day 42 after dietary modification. *P < 0.05, compared with the control group at each time point. ^†^*P* < 0.05, compared with day 7 in the same group (*n* = 5 for each group at each time point). Statistical analyses were performed with two-way ANOVA test followed by Tukey's test. HF, high-fat; HS, high-salt; HF+HS, high-fat with high-salt.

Compared to the normal diet, the HS diet or the HF+HS diet enhanced the intrarenal expression of proinflammatory cytokines/chemokines including TNF-α, INF-γ, MCP-1, and RANTES (Figure 4A). Expression of IL-6 was higher in the HF+HS diet group on day 7 (Supplemental Figure 3). Conversely, the intrarenal expression of VEGF in mice fed the HS diet and the HF+HS diet was significantly lower than that of the control group (Figure 4B).

### Dietary Modification Affects Susceptibility to Ischemic AKI

**Figure 5:**
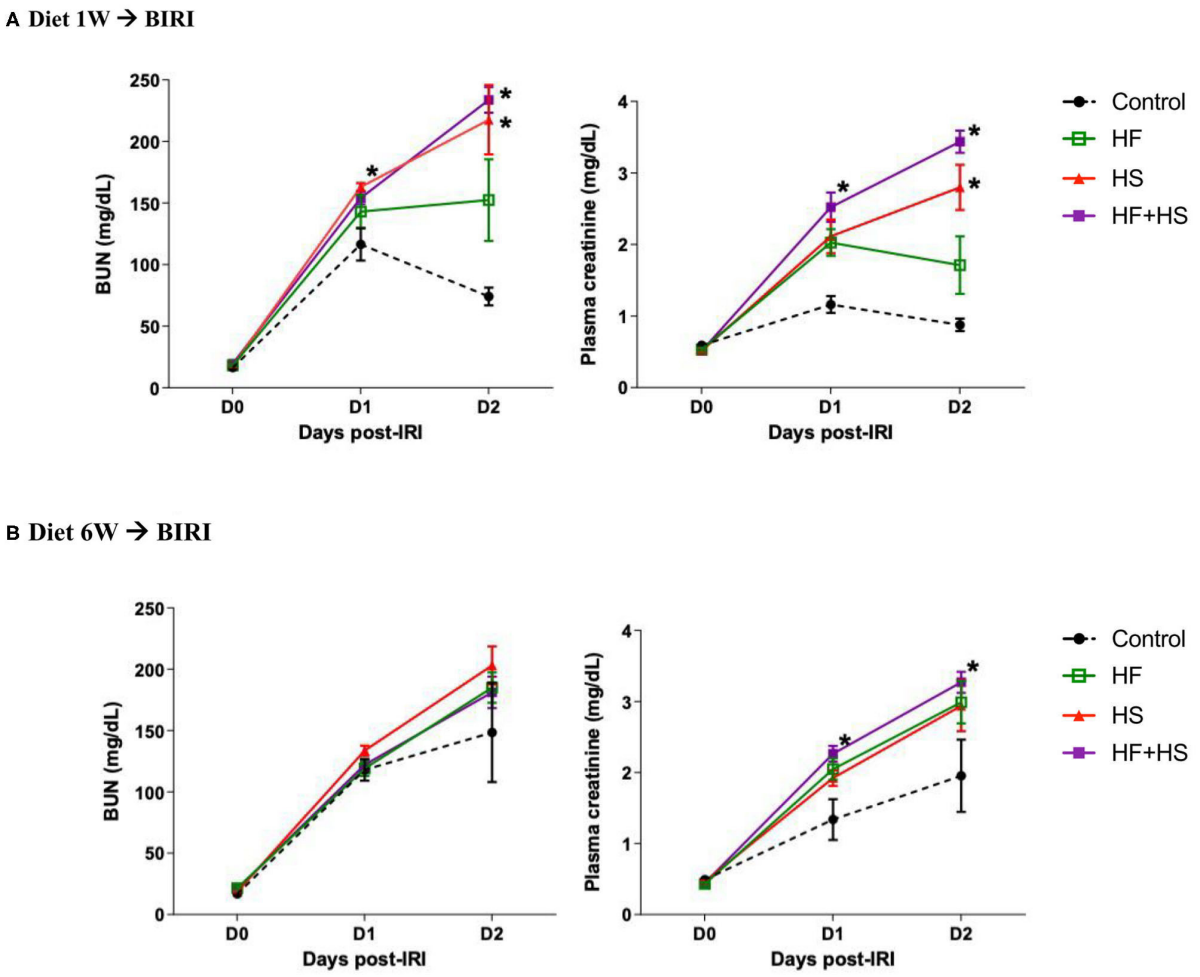
Effects of dietary modification on development of acute kidney injury (AKI) after bilateral IRI surgery. **(A,B)** The renal functional changes following IRI in each diet group. Deterioration of renal function following IRI tended to be aggravated by HF or a HS diet fed for both 1 week and 6 weeks. BUN and plasma creatinine levels were significantly higher in the HS and HF+HS diet groups fed for 1 week after IRI. Plasma creatinine level in the HF+HS diet group fed for 6 weeks was significantly higher than that of the control group on day 2 after IRI. *P < 0.05, compared with the control group at each time point (*n* = 5–10 for each group). Statistical analysis was performed using the Mann-Whitney *U*-test. HF, high-fat; HS, high-salt; HF + HS, high-fat with high-salt.

To investigate the effects of dietary modification on post-ischemic kidneys, bilateral IRI was performed 1 or 6 weeks after dietary modification. Overall, deterioration of renal function following IRI was more prominent in the mice receiving the HS-based diet modification for both 1 week and 6 weeks compared to the control group. Both BUN and plasma creatinine concentrations were significantly higher in the mice fed the HS or HF+HS diet for 1 week compared to the control group on day 2 after IRI (Figure 5A). Plasma creatinine concentration in the mice fed the HF+HS diet for 6 weeks was significantly higher than that in the control group on days 1 and 2 after IRI (Figure 5B).

**Figure 6:**
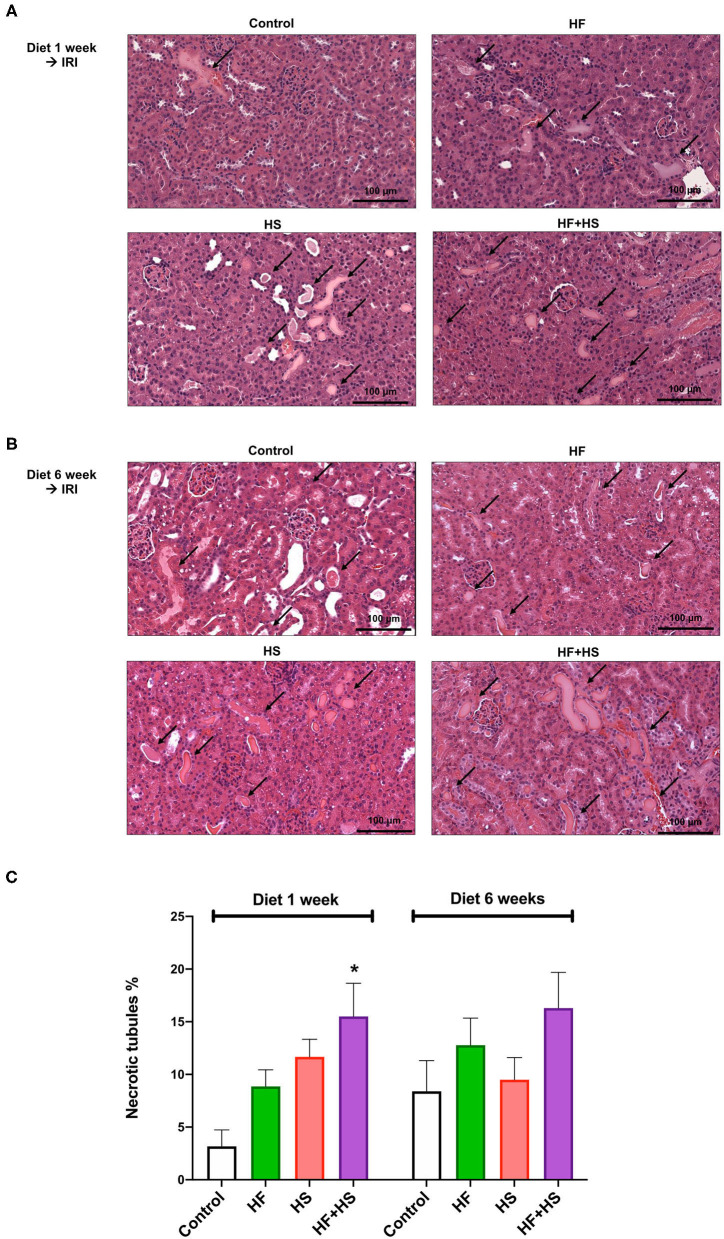
Effects of dietary modification on structural injury following IRI surgery. **(A,B)** Hematoxylin and eosin staining of post-ischemic kidneys on day 2 after IRI. Arrows indicate damaged or necrotic tubules (×200). **(C)** The percentages of necrotic tubules tended to be higher in the mice fed an HF or HS diet compared to the control group. The HF+HS diet group fed for 1 week showed a significantly higher percentage of necrotic tubules than the control group. *P < 0.05, compared with the control group (*n* = 5–10 for each group). Statistical analysis was performed using the Mann-Whitney *U*-test. HF, high-fat; HS, high-salt; HF + HS, high-fat with high-salt.

The proportions of necrotic tubules tended to be higher in the mice fed the HS or HF diet compared to the control group (Figure 6). The group fed an HF+HS diet for 1 week showed a significantly larger proportion of necrotic tubules than the control group (Figure 6C).

**Figure 7:**
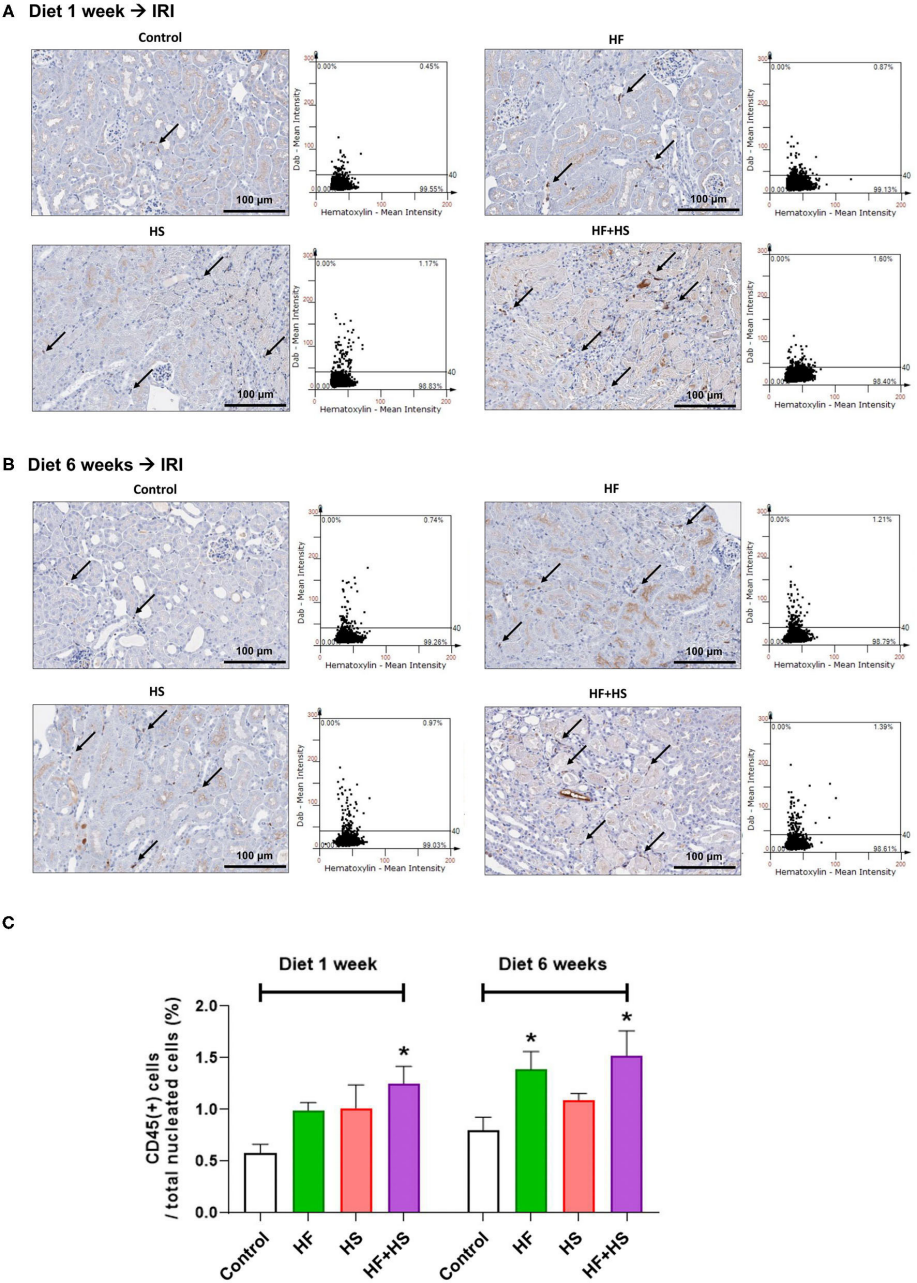
Effects of dietary modification on leukocyte trafficking into the post-ischemic kidney. **(A,B)** Representative immunohistochemistry findings and semiquantitative analyses of CD45-positive leukocytes in the post-ischemic kidneys on day 2 after IRI. There were more pronounced infiltrations of leukocytes into the post-ischemic kidneys of mice fed with an HF or HS diet for both 1 week and 6 weeks. Arrows indicate CD45-positive leukocytes (×200). **(C)** The percentages of total leukocytes expressing CD45 among total nucleated cells were higher in the post-ischemic kidneys of HF-or HS-fed mice compared with that of mice fed a normal diet. *P < 0.05, compared with the control group (*n* = 5–10 for each group). Statistical analysis was performed using the Mann-Whitney *U*-test. HF, high-fat; HS, high-salt; HF+HS, high-fat with high-salt.

The percentage of total leukocytes expressing CD45 among total nuclei in the post-ischemic kidneys was significantly higher in the group fed an HF+HS diet for both 1 week and 6 weeks (Figures 7A,B). The group fed an HF diet for 6 weeks also showed a larger proportion of CD45-positive cells than did the control group (Figure 7C).

**Figure 8:**
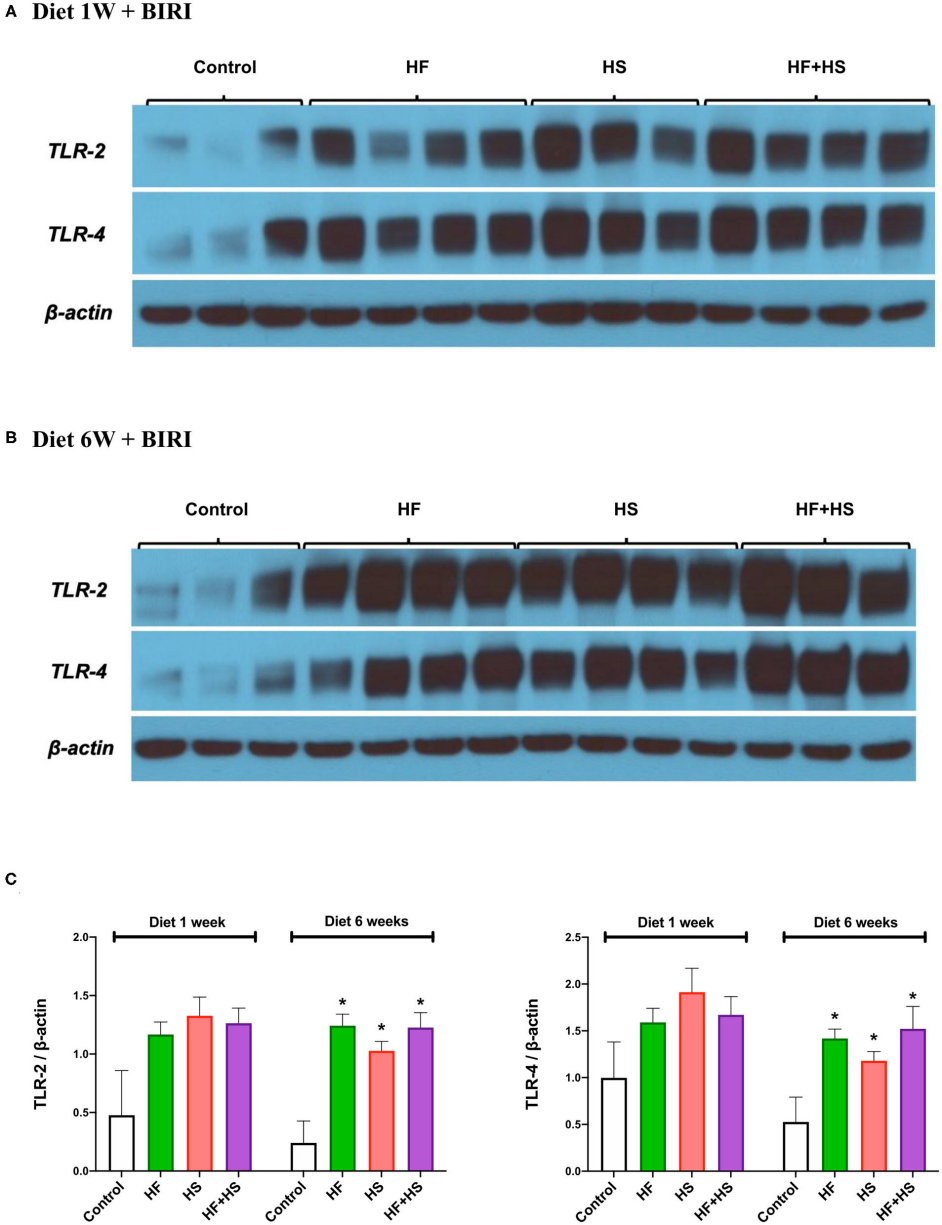
Western blotting of protein samples extracted from post-ischemic kidneys on day 2 after IRI. **(A)** Overall expression of TLR2 and TLR4 tended to be higher in post-ischemic kidneys of mice fed an HF or HS diet for 1 week compared to those of the mice fed a normal diet. **(B,C)** The expression of TLR-2 and TLR-4 was significantly higher in the HF, HS, and HF+HS diet groups fed for 6 weeks compared to the control group. *P < 0.05, compared with the control group (*n* = 3–4 for each group). Statistical analysis was performed using the Mann-Whitney *U*-test. HF, high-fat; HS, high-salt; HF+HS, high-fat with high-salt.

Intrarenal expression of TLR-2 and TLR-4 on day 2 following IRI was evaluated with western blotting of protein samples extracted from post-ischemic kidneys. The expression of both TLR-2 and TLR-4 tended to increase in mice fed HF or HS diet for 1 week (Figures 8A,C). Mice fed an HF+HS diet for 6 weeks showed significantly increased expression of both TLR-2 and TLR-4 (Figures 8B,C).

### Effects of High Sodium and Lipid on Hypoxic HK-2 Cells

**Figure 9:**
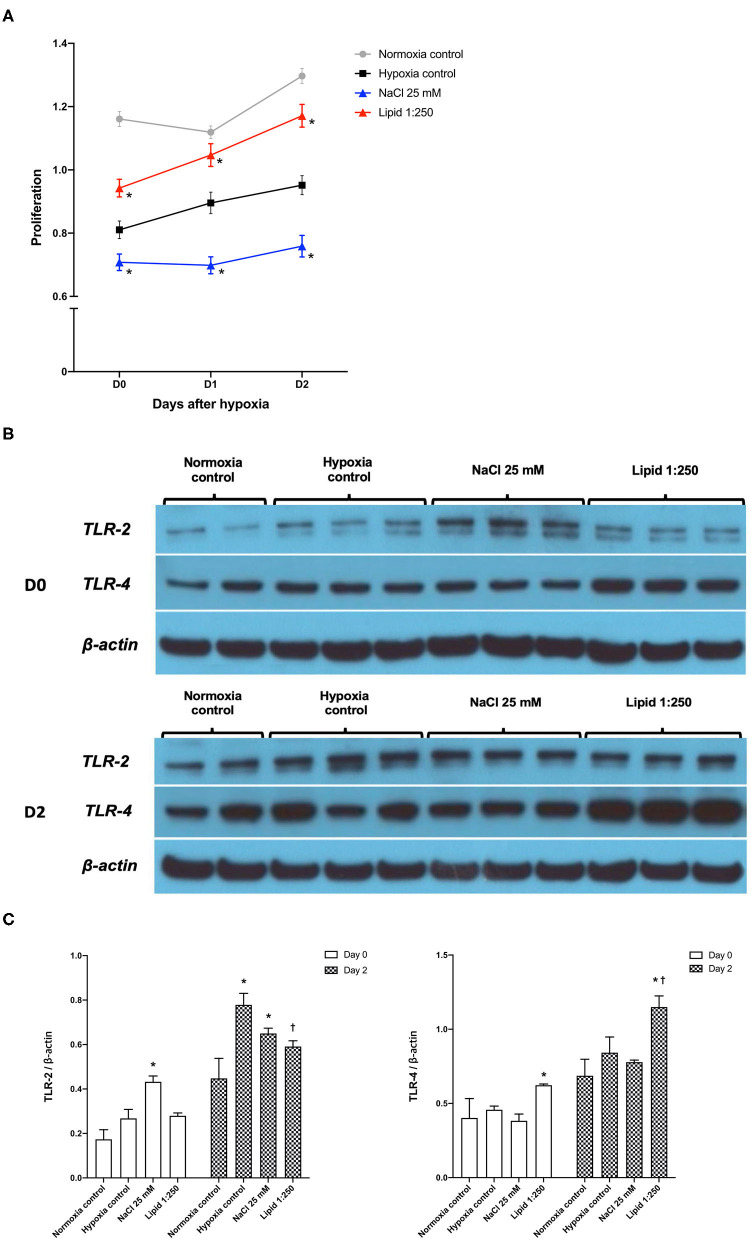
Effects of sodium and lipid treatment on hypoxic HK-2 cells. **(A)** Additional NaCl treatment inhibited the proliferation of HK-2 cells after hypoxic insult compared with the hypoxia control group. Conversely, additional lipid treatment facilitated the proliferation of HK-2 cells after hypoxic insult. **(B,C)** Western blotting of TLR-2 and TLR-4 showed that lipid treatment reduced the expressions of TLR-2 and enhanced the expression of TLR-4 compared with the hypoxia control group on day 2 after hypoxic insult. Day 0: Day when HK-2 cells were removed from the multi-gas incubator 48 h after hypoxia. *P < 0.05, compared with the normoxia control group. ^†^*P* < 0.05, compared with the hypoxia control group. Statistical analysis was performed using the Mann-Whitney *U*-test.

Figure 9A shows the degree of HK-cell proliferation after hypoxic insult depending on additional NaCl and lipid treatment. Day 0, the end of hypoxia, was the day when HK-2 cells were removed from the multi-gas incubator after 48 h of hypoxia. Treatment with high salt (additional NaCl 25 mM) suppressed the proliferation of hypoxic HK-2 cells. Conversely, lipid treatment facilitated the proliferation of hypoxic HK-2 cells.

Western blot analysis of TLR-2 and TLR-4 in protein extracts of hypoxic HK-2 cells showed that lipid treatment reduced the expression of TLR-2 and enhanced the expression of TLR-4, compared with the hypoxia control group on day 2 after hypoxic insult (Figures 9B,C). The additional NaCl treatment did not significantly change the expression of TLR-2 orTLR-4 compared to those of the hypoxia control group.

## Discussion

In this study, we investigated the effects of an HF or HS diet on normal kidneys and the development of ischemic AKI focusing on the intrarenal immunologic micromilieu in murine ischemic AKI and HK-2 cell hypoxia models. A HF or HS diet increased intrarenal CD8 T cells and plasma cells as well as changed intrarenal lymphocytes to more activated and mature phenotypes in normal kidneys. An HS diet increased intrarenal proinflammatory proinflammatory cytokines and decreased intrarenal anti-inflammatory cytokines. We also found variability in the intrarenal immunologic micromilieu by dietary modification in a diet switching experiment. However, effector memory CD8 T cells and TNF-α remained increased after returning to a normal diet. Therefore, an HF or HS diet seemed to induce a proinflammatory intrarenal immunologic micromilieu that was not completely reversible even after returning to a normal diet for at least 2 weeks. The deterioration of renal function following IRI was more prominent in the mice receiving a HS-based dietary modification before IRI. Dietary modification *per se* did not induce clinically apparent renal dysfunction. However, the proinflammatory changes in the intrarenal immunologic micromilieu caused by HF or HS diet seem to significantly increase susceptibility of renal injury following ischemic insult.

Inflammation plays an important role in the pathogenic mechanisms of AKI and CKD (6, 20–22). In addition to recruitment of circulating immune cells to kidneys, renal parenchymal cells and immune cells residing in the normal renal tissue comprise the intrarenal immunologic micromilieu (7, 22). Previous studies have demonstrated that environmental factors, such as commensal microbes or dietary composition, affect the immune system (9, 11, 12, 23). The effects of high levels of salt or fat on the intrarenal immune system have been studied in specific immune cells such as TH17 cells and regulatory T cells (24, 25), a salt-sensitive hypertensive model (11), and an HF diet-induced obesity model (12, 26). However, these studies have focused on only a few cellular components of the immune system or disease-specific experimental models and have provided a limited view of the complex effects of dietary modification. We evaluated the effects of HF or HS diet on the overall changes to the immunologic micromilieu of the normal kidney and reversibility after returning to a normal diet. Considering that there is no preventive management or effective treatment for AKI and transition of AKI to CKD, our study investigating the effects of dietary modification on susceptibility to ischemic AKI is important clinically.

Our study showed that dietary modification increased CD8 T cells and switched intrarenal T cells into effector memory subtypes, some of which persisted even after a normal diet for 2 weeks. These changes were most prominent in the group fed an HF+HS diet. T cells, especially CD4 T cells, play an important role in the early phase of ischemic AKI (23, 27). On the other hand, CD8 T cells did not appear to play a major role in the pathophysiology of AKI compared with CD4 T cells (28). Although the role of CD8 T cell has not been fully determined, production of IFN-γ by CD8 T cells increases in murine renal IRI and glomerulonephritis models, suggesting that CD8 T cells substantially contribute to the intrarenal inflammatory response in these diseases (29, 30).

During dietary modification, activated mature B cells increased at 2 weeks, and plasma cells increased at 6 weeks followed by decrement to level comparable with that of the control group after returning to a normal diet. These results suggest that an HF or HS diet can facilitate B cell activation and differentiation into plasma cells in normal kidneys. Furthermore, neutrophil and NK cells increased at 2 weeks after dietary modification and then decreased. As phenotype changes in T cells were observed mainly at 6 weeks, it can be hypothesized that HF and HS diets first activate the innate immune system and then activate the adaptive immune system. Previous studies have reported that B cells and plasma cells have pathogenic roles in post-ischemic kidneys (16, 31). Increased levels of intrarenal plasma cells by HF or HS diet seemed to promote renal injury after IRI in our study.

HF or HS diet also induced overall changes in intrarenal cytokines and chemokines of normal kidneys to proinflammatory conditions. IRI is known to stimulate the synthesis of proinflammatory cytokines such as TNF-α, IFN-γ, and IL-6 (32, 33) and chemokines such as MCP-1 (34, 35). Our results suggest that HF or HS diet can switch the intrarenal immunological micromilieu to a proinflammatory direction and subsequently enhance renal injury in ischemic AKI.

An HF or HS diet before IRI increased total CD45 T cells and TLR2 and TLR4 expression following IRI and induced more severe renal dysfunction during the early injury phase of IRI. This suggests that HF or HS diet enhances the innate immune response and aggravate IRI. It is worth mentioning that the increases in TLR4 expression and plasma creatinine in the group fed HF+HS diet were more remarkable compared with other groups, suggesting that HF and HS diets have additive effects on renal injury after IRI. In the HK-2 cell hypoxia model, HS treatment suppressed proliferation, but HS or high-lipid treatment did not show consistent effects on TLR expression. These differences of TLR expression in the *in vivo* and *in vitro* models suggest that the effects of dietary modification on the intrarenal immunologic micromilieu are complex responses based on the interaction between hypoxic tubular epithelial cells and intrarenal immune cells. The expression of TLR-2 and TLR-4 on renal tubular epithelial cells is enhanced in ischemic AKI (8), and TLR-2 contributes to renal injury after IRI as an important initiator of inflammatory responses (36). Therefore, our results further support more severe renal damage in ischemic AKI due to changes in the intrarenal immunologic micromilieu induced by dietary modification containing HF or HS compared to normal diet.

Interestingly, changes in the HF or HS diet were not consistent and some changes were offset, although the precise mechanisms of the different changes between the HF diet and the HS diet are unclear. For example, despite comparable amounts of dietary intake in the HF diet, HF+HD diet, and the normal diet group, body weight gain was more prominent in the HF diet group compared to the normal diet group, whereas HF+HS diet did not show this effect. The weight gain effect of the high fat component in the HF+HS group seemed to be offset by the high salt component. HS diet was previously reported to have an additive effect on the obesity induced by HF diet (37). However, weight gain of HF diet-fed mice was attenuated by HS diet in recent studies, which is consistent with the results of our study (38–40). One study suggested impaired digestive efficiency in the HF+HS group due to changes in renin-angiotensin system activity by HS (39), but another study did not find a difference in digestive efficiency between HF diet and HF + HS diet (40). Difference in cholesterol levels between the HF group and the HF+HS group showed a similar pattern. In addition, both the HF and HS groups promote proinflammatory immunologic micromilieu, but their effects on individual inflammatory cell or cytokine were slightly different. Although the molecular mechanisms of HS and HF on the immunologic micromilieu are not fully elucidated, different mechanisms of HF and HS diet, such as altered renal lipid metabolism and increased expression of sterol regulatory element-binding proteins in HF diet (41) and increased activity of intrarenal renin-angiotensin system in HS diet (25), have been reported to contribute to enhanced intrarenal inflammation. Therefore, the different molecular mechanisms of HS and HF diet induce different complex changes in intrarenal immunologic micromilieu. The HF diet and HS diet seem to have different effects on both physiologic changes and the intrarenal immunologic micromilieu. As shown in body weight changes in the HF+HS group, some immunologic effects including cellular and humoral components affected by HF or HS diet alone seemed to be counterbalanced by the other in the HF+HS group. Further study including comprehensive analysis of the effects of HF or HS diet on gut microbiome is required to reveal more precise effects of HF or HS diet on intrarenal immunologic changes and the underlying mechanisms of the interaction between HF diet and HS diet.

In our study, longer dietary modification did not show a greater negative effect on plasma creatinine concentration or renal pathologic findings. These were unexpected results since some intrarenal inflammatory cells and the expression of proinflammatory cytokines and TLR2/TLR4 were further increased after 6 weeks compared to 2 weeks of dietary modification. Physiologic adaptation limiting the negative effects of dietary modification might be a main reason for these findings, similar to the aldosterone escape phenomenon occurring in hyperaldosteronism (42). In addition, the effect of dietary modification in normal kidney was not constant over time. Some intrarenal inflammatory cells, such as activated mature B cells, neutrophils, and NK cells, increased at 2 weeks but decreased thereafter, and regulatory T cells increased at 6 weeks. It can be hypothesized that complex immunologic adaptation occurs, and different immunologic processes seem to be activated over time. It is well-known that the intensity of immunologic response can be decreased under hypo-responsive adaptation by low-level repeated stimulation, such as “LPS tolerance” (43). Our study suggested that even short-term dietary modification can adversely affect the course of AKI. Further studies are required to reveal more precise mechanisms of time-dependent intrarenal immunologic changes by dietary modification.

There are some limitations to this study. First, the precise mechanisms of time-dependent changes in some intrarenal immune cell populations in the control group were not fully elucidated. These changes might be caused by renal senescence since we previously reported that an NK T cell population tended to increase with aging (17). Second, the amount of salt used in the HS diet group was exceptionally large compared to human HS diet. Although the effects of HS diet depending on quantity and duration might be different, our study adequately simulated the long-term effects of moderate salt intake in humans using previously reported HS diet in murine models (44). Third, there was some discrepancy in TLR expression between the *in vivo* model and *in vitro* model. Further studies are required to investigate the complex interaction between intrarenal immune cells and injured tubular cells in post-ischemic kidneys.

In conclusion, this study demonstrated that dietary modification including HF or HS diet altered intrarenal immunologic micromilieu to proinflammatory condition and induced more severe renal injury following IRI. Our data can be used as the basis for recommendations to avoid HF or HS diet before surgery or procedure in kidney donors or patients who are at risk of ischemic AKI, such as heart failure patients.

## Data Availability Statement

The raw data supporting the conclusions of this article will be made available by the authors, without undue reservation.

## Ethics Statement

The animal study was reviewed and approved by Samsung Medical Center Animal Care and Use Committee.

## Author Contributions

HJ conceived and designed research. HJ, JJ, and KL performed experiments, analyzed data, interpreted results of experiments, prepared figures, and drafted manuscript. KY analyzed data. JL, GK, WH, DK, and Y-GK analyzed data, interpreted results of experiments, and revised the manuscript. All authors contributed to the article and approved the submitted version.

## Conflict of Interest

The authors declare that the research was conducted in the absence of any commercial or financial relationships that could be construed as a potential conflict of interest.

## Abbreviations

AKI: acute kidney injury
BIRI: bilateral ischemia-reperfusion injury
BUN: blood urea nitrogen
HK-2 cell: human kidney-2 cell
IFN-γ: interferon-γ
IL: interleukin
IRI: ischemia-reperfusion injury
KMNCs: kidney mononuclear cells
MCP-1: monocyte chemoattractant protein-1
RANTES: regulated on activation, normal T cell expressed and secreted
SEM: standard error of the mean
TLR: toll-like receptor
TNF-α: tumor necrosis factor-α
VEGF: vascular endothelial growth factor.
