# Supplementary material for: Dietary Modification Alters the Intrarenal Immunologic Micromilieu and Susceptibility to Ischemic Acute Kidney Injury
Source: Front Immunol. 2021 Mar 11;12:621176. doi: 10.3389/fimmu.2021.621176 (PMC7991094; doi:10.3389/fimmu.2021.621176)
Supplement: Supplementary file 1 [file Image_1.pdf]

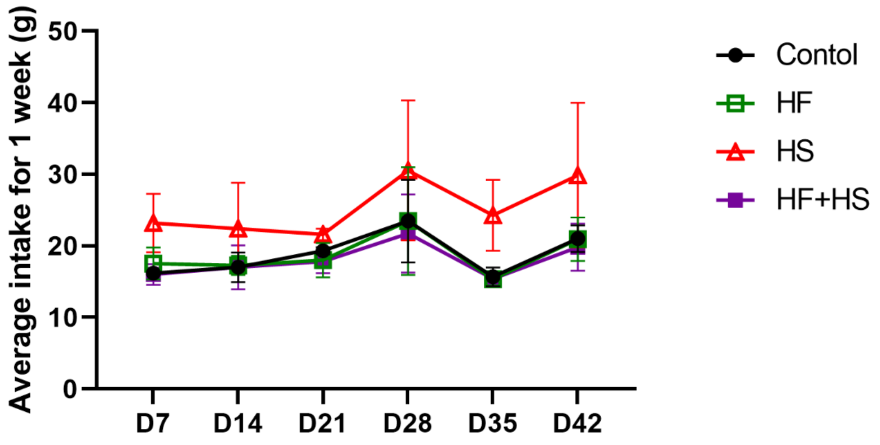

Supplemental Figure 1. Chow intake measured every 1 week during the dietary modification.
The total amount of dietary intake in the HF, HF+HS, and normal diet groups was similar. The HS group tended to consume slightly more chow. HF, high-fat; HS, high-salt; HF+HS, high-fat with high-salt.

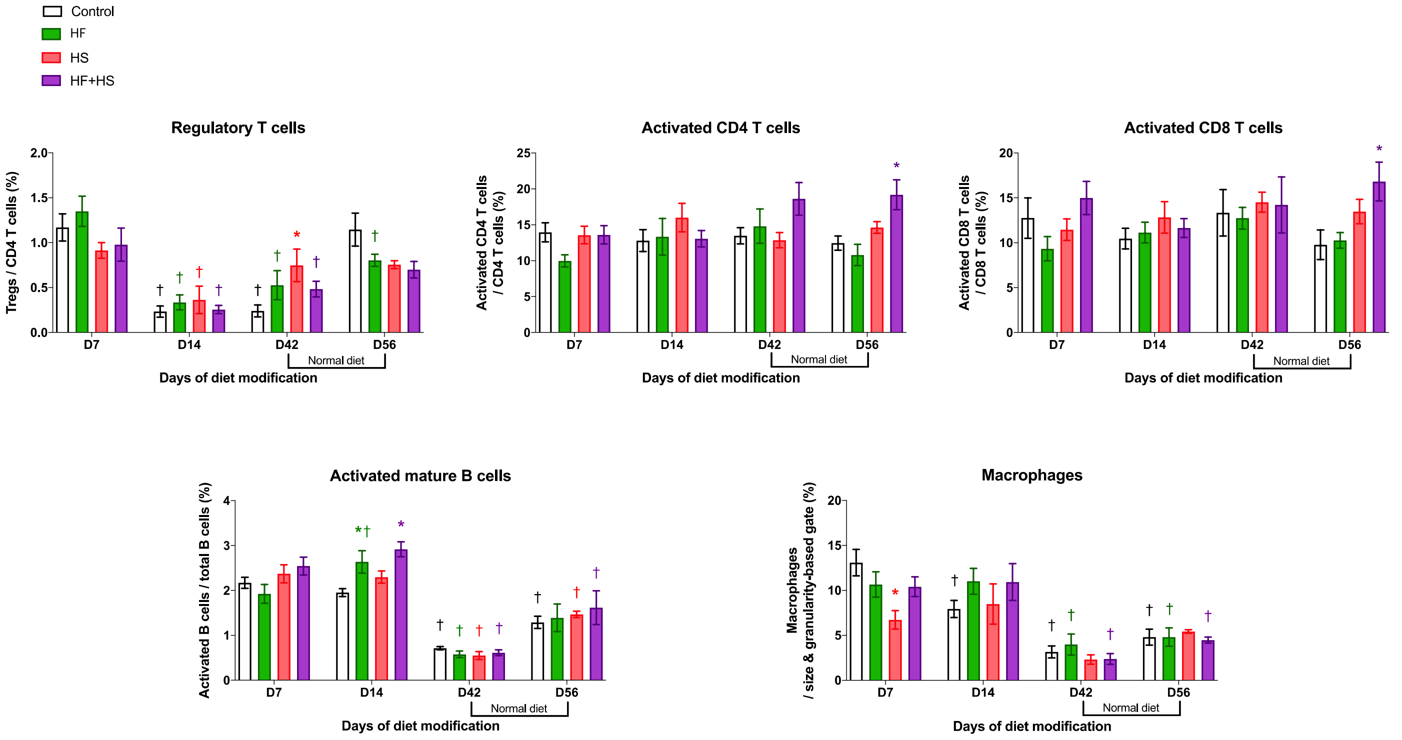

Supplemental Figure 2: Effects of dietary modification on leukocytes subtypes in kidneys of normal mice. Regulatory T cells among CD4 T cells were decreased over time from day 14 after the diet modification. The HS group showed higher expression of regulatory T cells compared to the control group on day 42. The HF+HS diet group showed higher proportion of activated phenotypes of CD4 and CD8 T cells compared to the control group on day 56. The proportions of activated mature B cells and macrophages were decreased from day 42. The HF and HF+HS diet groups showed facilitated infiltration of activated mature B cells on day 14.

*$P < 0.05$, compared with the control group at each time point. †$P < 0.05$, compared with day 7 in the same group ($n = 5$ for each group at each time point). Statistical analyses were performed with two-way ANOVA test followed by Tukey’s test. HF, high-fat; HS, high-salt; HF+HS, high-fat with high-salt.

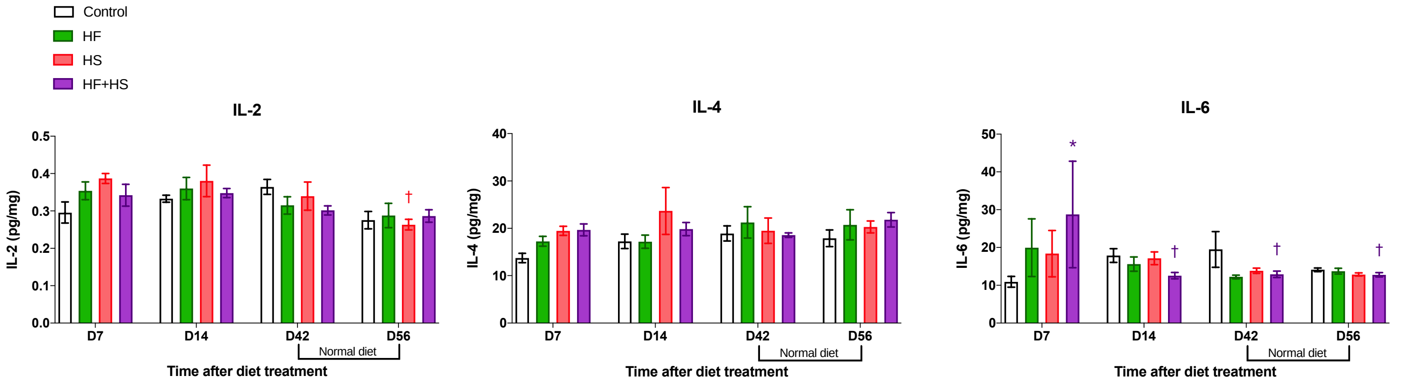

Supplemental Figure 3: Effects of dietary modification on IL-2, IL-4, and IL-6.
The expressions of IL-2 and IL-4 were comparable between the groups. The expression of IL-6 was higher in the HF+HS diet group on day 7 compared to the control group and decreased from day 14 after diet modification. **P* < 0.05, compared with the control group at each time point. †P< 0.05, compared with day 7 in the same group (*n* = 5 for each group at each time point). Statistical analyses were performed with two-way ANOVA test followed by Tukey's test. HF, high-fat; HS, high-salt; HF+HS, high-fat with high-salt.
